# The Impact of Aortic Calcification on Surgical Outcomes in Colorectal Cancer Patients: A Retrospective Analysis Focused on Anastomotic Leakage

**DOI:** 10.3390/medicina61040606

**Published:** 2025-03-27

**Authors:** Veysel Barış Turhan, Onur Karacif, Mehmet Berksun Tutan, Bahadır Kartal, Fatih Şahin, Murat Kendirci, Ertuğrul Gazi Alkurt

**Affiliations:** 1 Department of General Surgery, Faculty of Medicine, Hitit University, 19030 Çorum, Turkey; muratkendirci@gmail.com (M.K.); egalkurt@hotmail.com (E.G.A.); 2 Department of Radiology, Erol Olçok Training and Research Hospital, 19040 Çorum, Turkey; onurkaracif@gmail.com; 3 Department of General Surgery, Alaca State Hospital, 19600 Çorum, Turkey; mbtutan@gmail.com; 4 Department of General Surgery, Erol Olçok Training and Research Hospital, 19040 Çorum, Turkey; dr.bkartal@hotmail.com; 5 Department of General Surgery, Yüksekova State Hospital, 30110 Hakkari, Turkey; dr.fsahin@gmail.com

**Keywords:** anastomotic leak, aortic calcification, colorectal surgery, vascular health

## Abstract

*Background and Objectives*: Anastomotic leakage (AL) is a major complication of colorectal surgery (CRS), increasing morbidity, mortality, and healthcare costs. While several AL risk factors have been identified, the role of aortic calcification (AC) remains unclear. As a marker of systemic atherosclerosis, AC may impair tissue perfusion and anastomotic healing. Additionally, tumor factors (TNM stage, histology, and localization) and patient comorbidities (hypertension, cardiovascular disease, and neoadjuvant therapy) may contribute to AL risk. This study evaluates the association between preoperative AC and AL incidence while considering additional risk factors. *Materials and Methods*: This retrospective cohort study included 151 patients undergoing CRS from January 2020 to October 2023. Preoperative CT scans classified AC into Stage 0 (none), Stage 1 (<50%), and Stage 2 (>50%) of the aortic circumference. Data on demographics, tumor characteristics, neoadjuvant therapy, and comorbidities were collected. AL risk factors were analyzed using univariate and multivariate logistic regression. *Results*: AL occurred in 5.96% (9/151) of patients. AL incidence was significantly higher in patients with >50% AC (44.47% vs. 11.27%, *p* = 0.012). Multivariate analysis confirmed AC as an independent AL predictor (OR = 10.38, 95% CI: 1.243–92.118, *p* = 0.032). Rectal tumor localization (*p* = 0.038), hypertension (*p* = 0.027), cardiovascular disease (*p* = 0.014), and neoadjuvant therapy (*p* = 0.045) were also associated with increased AL risk. *Conclusions*: Severe AC is an independent predictor of AL in CRS. Additionally, rectal tumors, hypertension, cardiovascular disease, and neoadjuvant therapy contribute to AL risk. Preoperative vascular assessments and comprehensive risk stratification models may help identify high-risk patients and guide perioperative management strategies to reduce AL incidence.

## 1. Introduction

Colorectal surgeries (CRS) are becoming increasingly frequent today compared to previous years. As with all gastrointestinal surgeries, anastomotic leakage (AL) remains one of the most common and severe postoperative complications following CRS, significantly impacting morbidity, mortality, and healthcare costs [1]. The incidence of anastomotic leakage in colorectal surgeries varies between 3% and 19% in the literature, depending on surgical techniques, the patient’s overall health condition, and perioperative management approaches [2]. This poses an ongoing challenge for colorectal surgeons worldwide. Given the clinical and economic burden of AL, identifying risk factors that predispose patients to this complication is crucial for improving surgical outcomes and optimizing patient management [1].

One potential risk factor that has gained attention in recent years is aortic calcification (AC) [2,3]. AC is a marker of systemic atherosclerosis, reflecting widespread vascular disease and chronic inflammation. Since adequate tissue perfusion is vital for anastomotic healing, severe AC is hypothesized to contribute to microvascular dysfunction, ischemia at the anastomotic site, and ultimately, anastomotic healing failure [3]. Several studies have investigated the association between AC and AL in various gastrointestinal surgeries, including esophagectomy and colorectal resections, reporting conflicting findings [3,4,5,6,7].

In addition to vascular factors such as AC, tumor-related characteristics and patient comorbidities may also influence AL risk. Tumor histology, TNM stage, tumor localization, and neoadjuvant oncological therapy have been implicated as potential contributors to anastomotic healing failure [8]. Moreover, comorbidities such as hypertension, diabetes mellitus, and cardiovascular disease are known to impair vascular integrity and wound healing, thereby further increasing the risk of AL [9]. However, there is a limited number of studies that systematically evaluate these variables in conjunction with aortic calcification as risk factors for AL. Furthermore, the role of nutritional status in anastomotic healing is increasingly recognized, particularly in colorectal cancer patients who often present with sarcopenia due to underlying malignancy. Malnutrition is associated with impaired wound healing and increased risk of postoperative complications. Therefore, multidisciplinary teams should incorporate nutritional experts to optimize perioperative care [10].

This study introduces an innovative approach by evaluating the impact of aortic calcification (AC) as a potential predictor of anastomotic leakage in colorectal surgery patients. AC, as a marker of systemic atherosclerosis, is hypothesized to impair microvascular perfusion and negatively affect anastomotic healing. While previous studies have assessed the relationship between vascular disease and AL, the specific role of AC in colorectal surgery remains unclear. Our study aims to analyze AC in conjunction with oncological and patient-related risk factors to provide a comprehensive risk stratification framework that may enhance perioperative decision-making.

## 2. Materials and Methods

The study period spanned from January 2020 to October 2023. During this time, patients diagnosed with colorectal carcinoma and operated on by the Department of General Surgery at Erol Olçok Training and Research Hospital were evaluated systematically for this study. Since this study involved a retrospective analysis of anonymized patient data, the requirement for informed consent was waived. The surgical procedures were performed by a team of experienced colorectal surgeons, each with a minimum of 10 years of specialized surgical experience. The center where the study was conducted performs an average of 250 colorectal cancer surgeries per year, ensuring a high-volume setting conducive to robust clinical assessment.

The study adhered to the STROBE (Strengthening the Reporting of Observational Studies in Epidemiology) checklist to ensure rigorous methodological reporting and transparency. All relevant observational study components were assessed and included where applicable. The inclusion criteria were as follows: patients aged between 18 and 90 years; diagnosed with colorectal carcinoma and undergoing colorectal surgery with primary anastomosis; availability of preoperative CT scans for aortic calcification assessment; and no known hematological, oncological, or severe vascular diseases apart from AC. The exclusion criteria: patients with incomplete medical records, pre-existing hematological malignancies, or other severe vascular diseases affecting the study variables.

Additionally, only patients who underwent elective surgeries were included in the study. Patients who underwent emergency colorectal surgery were excluded, as emergency surgery has been shown to increase the risk of anastomotic leakage.

A power analysis was conducted to determine the statistical power of the study. Based on a significance level of *p* < 0.05, 80% power (1 − β), and the expected effect size, at least 86 patients were required. The final sample size of 151 patients met this requirement and was included in the study after applying the aforementioned exclusion criteria. Patients were consecutively enrolled based on the availability of complete preoperative imaging and clinical records.

### 2.1. Data Collection

In the retrospective analysis, data regarding patient demographics (age and gender), tumor localization, aortic calcification (AC) measurements, and anastomotic leak (AL) incidents were systematically collected. Additionally, tumor histology (adenocarcinoma, mucinous carcinoma, other), TNM staging (I, II, III, IV), neoadjuvant oncological therapy (chemotherapy and/or radiotherapy), and comorbidities (hypertension, diabetes mellitus, cardiovascular disease, smoking history) were recorded from patient medical records.

Patients were further divided into two subgroups based on tumor localization: colon cancer (*n* = 102) and rectal cancer (*n* = 49). Anastomotic leakage incidence was analyzed separately for each group. Among rectal cancer patients, AL occurred in 2 cases (4.08%), while in the colon cancer group, AL was observed in 7 cases (6.86%). Statistical comparisons between the two subgroups were performed to determine if tumor localization played a significant role in AL development.

### 2.2. Anastomotic Leak Diagnosis Protocol

Anastomotic leakage (AL) was diagnosed based on a combination of clinical, radiological, and intraoperative findings:

- Clinical indicators: abdominal pain, fever, peritonitis, or increased inflammatory markers.
- Drain output analysis: presence of fecaloid or high-amylase content in the drain fluid.
- Radiological evidence: computed tomography (CT) scans showing anastomotic extravasation, perianastomotic fluid collection, or abscess formation.

### 2.3. Ethical Approval and Patient Consent

This study was designed retrospectively and received ethical approval from the Institutional Review Board of the Hitit University Faculty of Medicine Clinical Research Ethics Committee (Protocol Number: 2023-143, date: 1 November 2023). Since this was a retrospective study with anonymized patient data, informed consent was waived.

### 2.4. Imaging Protocol and Calcification Measurement

Preoperative computed tomography (CT) scans were evaluated by two independent specialists blinded to surgical outcomes. Standard imaging parameters included a tube current of 150 mA, tube voltage of 120 kVp, section thickness of 5 mm, and section interval of 5 mm. The classification of aortic calcification (AC) was standardized using a predefined grading system.

**Figure 1:**
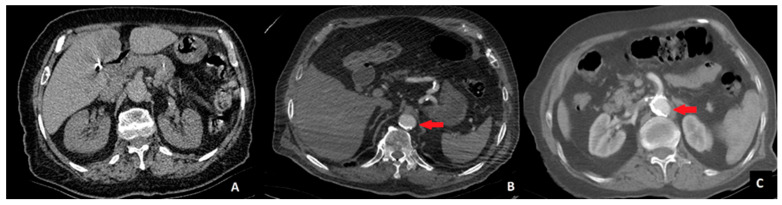
Computed tomography images of patients. **A**—no calcification (Stage 0), **B**—calcified area less than 50% of the aortic circumference (Stage 1), and **C**—calcified area more than 50% of the aortic circumference (Stage 2). The area of calcification is indicated by a red arrow.

To ensure consistency, radiologists independently assessed all scans, and interobserver agreement was measured using Cohen’s kappa coefficient (κ). In cases of disagreement, a consensus meeting was conducted to finalize classification. This approach minimized variability and improved reliability in AC grading. Aortic calcification was assessed in the segment between the celiac trunk and the aortic bifurcation using axial and coronal CT images. Aortic calcification was classified into three stages based on the extent of calcified plaques encircling the aortic lumen. Stage 0 was defined as the absence of any detectable calcification. Stage 1 was assigned when calcification occupied less than 50% of the aortic circumference. Stage 2 was defined as calcification occupying more than 50% of the aortic circumference. A visual example of each calcification stage is provided in Figure 1.

### 2.5. Statistical Analysis

All statistical analyses were conducted using IBM SPSS Statistics for Windows software (version 26; IBM Corp., Armonk, NY, USA). Descriptive statistics were reported for categorical variables as counts and percentages and for numerical variables as mean ± standard deviation or median ± interquartile range based on data distribution.

The normality of data distribution was assessed using the Shapiro–Wilk test. Correlations between variables were evaluated using Pearson and Spearman correlation coefficients depending on data distribution. Comparisons of numerical variables between groups, such as age, were made using the Student’s *t*-test or Mann–Whitney U test, based on the normality assumption. Categorical variables, including gender, tumor localization, histology, TNM stage, comorbidities, neoadjuvant therapy, and AC incidence, were evaluated for differences between groups using the Chi-square test or Fisher’s exact test.

A multivariate logistic regression analysis was performed to adjust for potential confounding factors. The model included variables such as age, sex, TNM stage, tumor histology, tumor localization, neoadjuvant therapy, hypertension, diabetes mellitus, cardiovascular disease, smoking, and intraoperative hypotension. The interaction between AC and AL was assessed using univariate binomial logistic regression analysis with bootstrapping. The odds ratio (OR) of aortic calcification for developing anastomotic leakage was calculated, and a *p*-value of <0.05 was considered statistically significant in all analyses.

## 3. Results

A total of 151 patients were included in the study cohort, with a mean age of 65.39 ± 10.97 years. Among them, 93 (61.59%) were male, and 58 (38.41%) were female. The most prevalent tumor localization was the rectum (32.45%), followed by the ascending colon (31.13%) (Table 1).

Among the patients, 126 (83.44%) had adenocarcinoma, 19 (12.58%) had mucinous carcinoma, and 6 (3.97%) had other histological subtypes. Based on TNM staging, 17 (11.25%) patients were Stage I, 52 (34.44%) were Stage II, 61 (40.39%) were Stage III, and 21 (13.91%) were Stage IV. No significant differences were observed in histological subtypes between the AL and non-AL groups (*p* = 0.438), whereas higher TNM stages (Stage III/IV) were slightly more frequent in the AL group; however, the difference did not reach statistical significance (*p* = 0.089) (Table 2).

**Figure 2:**
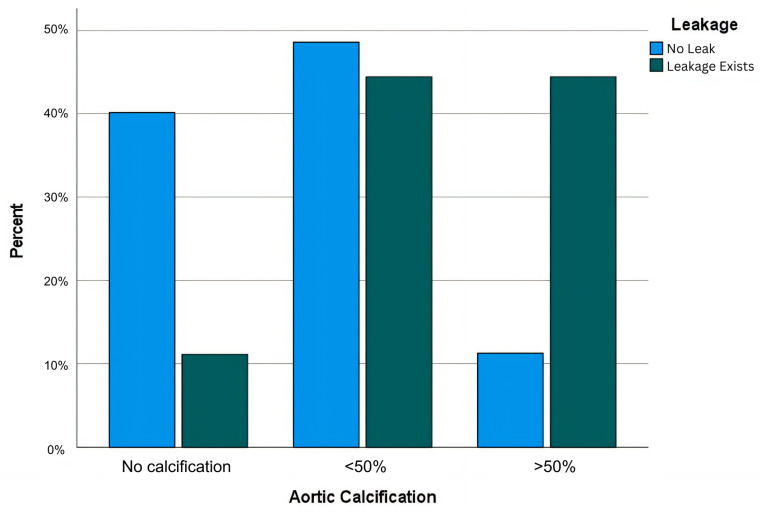
Anastomotic leakage percentages between aortic calcification groups.

**Figure 3:**
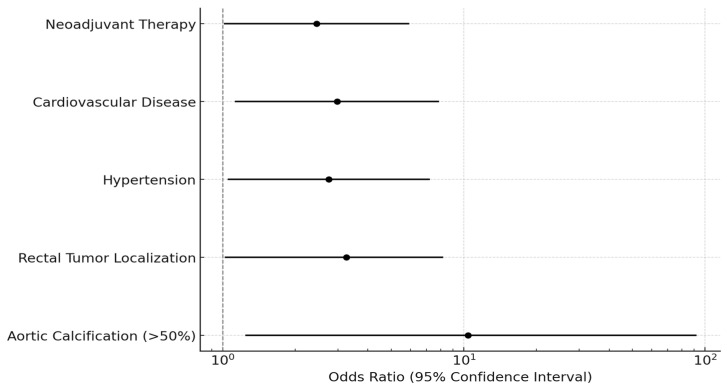
Forest plot of risk factors for anastomotic leak.

Comorbidities were prevalent among the study population, with hypertension (HT) in 68 patients (45.03%), diabetes mellitus (DM) in 42 (27.81%), cardiovascular disease (CVD) in 31 (20.53%), and smoking history in 47 (31.12%) patients. Patients in the AL group had a higher prevalence of HT (66.67% vs. 43.66%, *p* = 0.027) and CVD (44.44% vs. 18.30%, *p* = 0.014) compared to the non-AL group. Additionally, 38 (25.17%) patients received neoadjuvant oncological therapy (chemotherapy and/or radiotherapy), and the AL incidence was higher in patients who received neoadjuvant treatment (10.52% vs. 4.18%, *p* = 0.045). Anastomotic leakage percentages are displayed in Figure 2. To enhance the visualization of risk factor contributions, a forest plot illustrating the odds ratios (OR) of significant variables influencing anastomotic leakage (AL) has been included as Figure 3.

38.41% of the patients exhibited no calcification (Stage 0), 48.34% had calcification occupying less than half of the aortic circumference (Stage 1), and 13.25% had calcification occupying more than half of the aortic circumference (Stage 2). Anastomotic leaks were observed in 9 out of 151 patients (5.96%) postoperatively. When patients were stratified based on the presence or absence of AL, there were no significant differences in mean age (*p* = 0.543) or gender distribution (*p* = 0.747). However, a notable discrepancy was observed in tumor localization, as rectal tumors had a significantly higher AL incidence compared to colonic tumors (8.3% vs. 4.2%, *p* = 0.038). Additionally, a significant difference in AC prevalence was observed between the leakage and no-leakage groups, with a higher prevalence of >50% AC in the leakage group (44.47%) compared to the no-leakage group (11.27%, *p* = 0.012).

**Figure 4:**
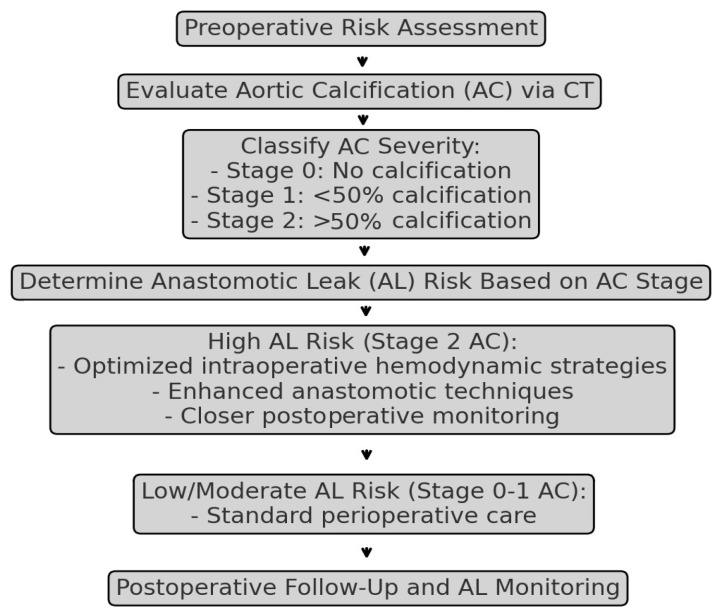
Graphical algorithm summarizing the risk assessment and management strategy for anastomotic leak (AL) based on aortic calcification (AC).

Further analysis employing binomial logistic regression with bootstrapping revealed that patients with >50% AC exhibited a substantially elevated risk of AL. The odds ratio (OR) for developing AL in patients with >50% AC was 14.25 [95% CI: 1.486–136.611], with a *p*-value of 0.021 indicating statistical significance (Table 3). Conversely, multivariate analysis adjusting for TNM stage, hypertension, cardiovascular disease, and neoadjuvant therapy showed that AC remained an independent risk factor for AL (OR = 10.38, 95% CI: 1.243–92.118, *p* = 0.032). These findings underscore the potential association between extensive AC and an increased risk of AL following CRS. Additionally, rectal tumor localization, hypertension, cardiovascular disease, and neoadjuvant therapy were associated with increased AL risk. A graphical algorithm summarizing the risk assessment and management strategy for anastomotic leak (AL) based on aortic calcification (AC) severity has been developed and is included as Figure 4.

## 4. Discussion

Despite advances in clinical experience and the development of advanced surgical tools, anastomotic leakage (AL) remains one of the most serious complications in colorectal cancer surgery. Although various studies have aimed to elucidate the relationship between arteriosclerosis and AL in CRS, the associations remain unclear. Our study demonstrates a significant correlation between the extent of aortic calcification (AC), as assessed by preoperative CT, and the risk of AL. Specifically, patients with >50% AC were found to have a markedly higher risk of AL, highlighting the clinical importance of preoperative vascular assessments in colorectal surgeries.

Several previous studies have analyzed the predictive value of AC for AL. Deguelte et al. reported that abdominal aortic calcification ≥ 5% should be indicative of a higher risk of AL [11]. Eveno et al.’s study also determined that aortic artery calcification was an independent determinant of postoperative anastomotic fistula in patients undergoing left colon or rectum resection [12]. These findings parallel our results and suggest that measuring aortic artery calcification in risk assessment before CRS may enhance risk prediction accuracy. Knight et al. and Morita et al. also confirmed that abdominal aortic calcification could be an indicator of AL [13,14]. Previous studies have reported that abdominal aortic calcification (AC) may increase the risk of AL development [11,12]. Our study supports this literature, demonstrating that severe AC is an independent risk factor for AL. However, the study by Pochhammer et al. reported that AC is not directly associated with AL. These conflicting results highlight the need for larger-scale studies [15]. However, Pochhammer et al. argued that impairment of visceral circulation did not affect CRS outcomes, especially in the presence of AL. The discrepancies between our findings and those of Pochhammer et al. may be attributed to differences in study design, patient selection criteria, and AC measurement methods. While our study utilized a systematic classification of AC severity based on preoperative CT scans, Pochhammer et al. primarily assessed iliac artery calcifications, which may have different implications for colonic microcirculation. Furthermore, variations in surgical techniques, patient demographics, and perioperative care protocols across studies may contribute to these conflicting results.

Our study suggests that the association between AC and AL may be due to atherosclerosis and its impact on tissue perfusion. Endothelial dysfunction, associated with reduced nitric oxide production, may contribute to arterial constriction and diminished microvascular circulation at the anastomotic site. This may impair oxygen delivery and delay essential wound healing processes, such as fibroblast proliferation and collagen deposition. Additionally, arterial stiffness may impair adaptive hemodynamic responses, leading to increased anastomotic vulnerability in patients with severe vascular calcification [16,17]. When AC occurs, arterial narrowing limits blood flow to the anastomotic site, thereby increasing the risk of AL due to compromised tissue healing [18,19,20]. Ischemic changes, as predictors of AL, have also been reported by Vignali et al., supporting our hypothesis [21].

Given the findings of our study, certain intraoperative strategies may help mitigate AL risk in patients with significant aortic calcification: selective use of near-infrared fluorescence angiography to assess anastomotic perfusion intraoperatively; modified anastomotic techniques, including reinforcement suturing or tension-free anastomoses; optimized hemodynamic management, particularly in high-risk vascular patients to prevent transient hypoperfusion postoperatively; avoiding excessive bowel traction and ensuring minimal anastomotic tension to promote better perfusion; and proximal diversion stomas could be considered in high-risk cases to allow better anastomotic healing.

In addition to AC, our study identified several other factors associated with an increased risk of AL. Rectal tumor localization was found to have a higher incidence of AL compared to colonic tumors. This is consistent with previous studies suggesting that rectal anastomoses may be more vulnerable due to lower blood supply and increased technical complexity [22]. Furthermore, patients with hypertension and cardiovascular disease exhibited significantly elevated AL rates, suggesting that systemic vascular health plays a crucial role in anastomotic healing.

The strong association between extensive AC and AL emphasizes the necessity for comprehensive vascular assessments in patients scheduled for CRS. Identifying high-risk patients with significant AC enables the adoption of tailored perioperative strategies to potentially mitigate AL risk. For instance, enhanced cardiovascular interventions and vigilant postoperative monitoring could be beneficial in this patient subset [23]. Given the multifactorial nature of AL, integrating AC severity, TNM staging, tumor localization, and patient comorbidities into a comprehensive risk stratification model could enhance preoperative planning. A predictive scoring system incorporating these variables may help identify high-risk patients who could benefit from targeted interventions, such as preoperative vascular optimization, perioperative hemodynamic monitoring, and modified anastomotic techniques.

Moreover, the subgroup analysis revealed that rectal cancer patients exhibited a slightly lower AL incidence than colon cancer patients (4.08% vs. 6.86%). While this difference was not statistically significant, it underscores the necessity of further investigations to determine whether different tumor locations impact AL risk differently. Several factors, including the technical complexity of rectal anastomoses, reduced perfusion in low rectal regions, and differences in neoadjuvant therapy, may contribute to this trend. Future research should focus on prospective studies to elucidate whether preoperative vascular assessments should be tailored to different tumor locations.

Our study has several limitations. First, due to its retrospective design, there is a potential risk of selection bias and information bias. As data collection relies on medical records, there is a possibility that some clinical variables were misreported or missing. To mitigate this limitation, patient medical records were systematically reviewed, and missing data were minimized. However, prospective studies are needed to validate these findings more robustly. Second, the assessment of aortic calcification (AC) was performed manually, which may introduce interobserver variability. Manual measurements may lead to subjective differences between observers, potentially affecting the consistency of the results. To minimize this bias, future studies should consider using automated image analysis systems or artificial intelligence-assisted measurement techniques to improve accuracy and reproducibility. Third, the relatively small sample size may limit the statistical power of the study. The low incidence of anastomotic leak (AL) could influence the significance level of certain variables in statistical analyses. Particularly in multivariate analyses, larger patient cohorts are required to better establish independent risk factors. Future multi-center prospective studies will be essential to validate these associations. Lastly, there may be potential confounding factors that were not accounted for in this study. Factors such as smoking status, diabetes, and hypertension are known to influence vascular health and could have a direct impact on AL risk. While our study attempted to control for major variables, we acknowledge that additional multivariate analyses with a broader range of variables could further clarify whether AC is an independent risk factor.

Despite these limitations, this study has several strengths. Comprehensive risk factor analysis: it evaluates the association between aortic calcification and anastomotic leakage while adjusting for multiple confounders, including tumor characteristics and comorbidities. Retrospective cohort design with detailed subgroup analysis: the inclusion of both colon and rectal cancer patients allows for a better understanding of tumor localization’s impact on AL risk. Standardized imaging assessment: aortic calcification evaluation was performed systematically with an independent radiologist review to enhance data reliability. Exclusion of emergency cases: by focusing on elective surgeries, we minimized confounding factors associated with emergency interventions.

Additionally, our study was conducted in a single high-volume center, which may limit generalizability to lower-volume hospitals. Future prospective, multicenter studies with larger sample sizes will be required to validate these findings and provide a more comprehensive understanding of AC’s role in AL development. Moreover, further research should explore the potential role of intraoperative tissue perfusion monitoring and its correlation with vascular calcifications in predicting AL risk.

Recent research has highlighted the role of gut microbiota in anastomotic healing, with alterations in intestinal microbiota potentially contributing to increased AL risk. Preoperative treatments, including probiotics or dietary modifications, may influence microbial balance and improve anastomotic integrity [24].

As a practical recommendation, preoperative vascular risk assessment protocols should be considered for patients undergoing colorectal surgery. Patients with extensive aortic calcification (>50%) may benefit from optimized intraoperative hemodynamic strategies, enhanced anastomotic techniques, or closer postoperative monitoring to mitigate AL risk. Additionally, dysmetabolic patients may experience transient hypovascularization in the early postoperative period if not supported with inotropes, potentially increasing anastomotic leakage risk. Maintaining hemodynamic stability in these patients should be a key consideration in perioperative care.

After inferior mesenteric artery ligation, only the marginal artery provides blood supply to the proximal anastomotic stump. Surgeons should be aware of this vascular limitation, particularly in patients with pre-existing vascular disease.

## 5. Conclusions

In conclusion, our study highlights a significant association between extensive aortic calcification and an increased risk of AL following CRS. Additionally, rectal tumor localization, hypertension, cardiovascular disease, and neoadjuvant therapy were associated with increased AL risk. Preoperative vascular assessments, along with comprehensive risk stratification models incorporating oncological and systemic factors, could help identify patients at higher risk, allowing for the implementation of tailored perioperative strategies. Future studies with larger sample sizes and a prospective design are needed to further elucidate the role of AC in AL and validate these findings.

## Figures and Tables

**Table 1:** Demographic specifications of the patients and comparison between the patient groups.

Variables		All Patients (*n* = 151)	No-Leak (*n* = 142)	Leakage (*n* = 9)	Statistical Significance
Age		65.39 ± 10.97	65.25 ± 11.07	67.56 ± 9.41	0.543
Gender	Female	58 (38.41%)	55 (38.73%)	3 (33.33%)	0.747
	Male	93 (61.59%)	87 (61.27%)	6 (66.67%)	
Localization	Ascending colon	47 (31.13%)	44 (30.99%)	3 (33.33%)	0.815
	Transverse colon	4 (2.65%)	4 (2.82%)	0 (0%)	
	Splenic flexure	9 (5.96%)	9 (6.34%)	0 (0%)	
	Descending colon	7 (4.64%)	6 (4.23%)	1 (11.11%)	
	Sigmoid colon	28 (18.54%)	26 (18.31%)	2 (22.22%)	
	Rectosigmoid	7 (4.64%)	6 (4.23%)	1 (11.11%)	
	Rectum	49 (32.45%)	47 (33.1%)	2 (22.23%)	
Aortic Calcification	No calcification	58 (38.41%)	57 (40.14%)	1 (11.11%)	0.012
	<50%	73 (48.34%)	69 (48.59%)	4 (44.44%)	
	>50%	20 (13.25%)	16 (11.27%)	4 (44.47%)	

**Table 2:** Tumor characteristics, comorbidities, and neoadjuvant therapy in patients undergoing colorectal surgery.

Variables	All Patients (*n* = 151)	No-leak (*n* = 142)	Leakage (*n* = 9)	*p*-Value
Histology–Adenocarcinoma (%)	126 (83.44%)	118 (83.1%)	8 (88.89%)	-
Histology–Mucinous carcinoma (%)	19 (12.58%)	18 (12.68%)	1 (11.11%)	-
Histology–Other (%)	6 (3.97%)	6 (4.22%)	0 (0%)	-
TNM Stage–I (%)	17 (11.25%)	15 (10.56%)	2 (22.22%)	0.089
TNM Stage–II (%)	52 (34.44%)	49 (34.51%)	3 (33.33%)	
TNM Stage–III (%)	61 (40.39%)	58 (40.85%)	3 (33.33%)	
TNM Stage–IV (%)	21 (13.91%)	20 (14.08%)	1 (11.11%)	
Comorbidities– Hypertension (%)	68 (45.03%)	62 (43.66%)	6 (66.67%)	0.027
Comorbidities– Diabetes Mellitus (%)	42 (27.81%)	40 (28.17%)	2 (22.22%)	-
Comorbidities– Cardiovascular Disease (%)	31 (20.53%)	26 (18.30%)	4 (44.44%)	0.014
Comorbidities– Smoking (%)	47 (31.12%)	N/A	N/A	-
Received Neoadjuvant Therapy (%)	38 (25.17%)	6 (4.18%)	4 (10.52%)	0.045

**Table 3:** Binomial logistic regression test results for aortic calcification stages.

Variables	Wald	Sig.	Exp(B) [OR]	95% CI for OR
No Aortic Calcification	6.699	0.035		
Aortic Calcification < 50%	1.114	0.291	3.304	0.359–30.400
Aortic Calcification > 50%	5.307	0.021	14.25	1.486–136.611
Constant	16.064	<0.001	0.018	

OR: odds ratio, CI: confidence interval.

## Data Availability

The data presented in this study are available on reasonable request from the corresponding author due to privacy.

## Abbreviations

The following abbreviations are used in this manuscript:

AL	Anastomotic leakage
AC	Aortic calcification
CRS	Colorectal surgery
CT	Computerized tomography
CI	Confidence interval
OR	Odds ratio
